# Quantitative analysis of myelinic fibers in human laryngeal nerves according to age

**DOI:** 10.1016/S1808-8694(15)30750-3

**Published:** 2015-10-19

**Authors:** Romualdo Suzano Louzeiro Tiago, Paulo Augusto de Lima Pontes, Osíris de Oliveira Camponês do Brasil

**Affiliations:** 1 PhD in Sciences – Graduate Program in Otolaryngology and Head and Neck Surgery – Federal University of São Paulo - Escola Paulista de Medicina. MD. Researcher at the Department of otorhinolaryngology and Head and Neck Surgery – Federal University of São Paulo - Escola Paulista de Medicina; 2 Associate Professor of Otorhinolaryngology and Head and Neck Surgery – Federal University of São Paulo - Escola Paulista de Medicina. Full Professor – Department of otorhinolaryngology and Head and Neck Surgery – Federal University of São Paulo - Escola Paulista de Medicina; 3 PhD in Medicine – Federal University of São Paulo - Escola Paulista de Medicina. Professor at the Graduate Program - Department of otorhinolaryngology and Head and Neck Surgery – Federal University of São Paulo - Escola Paulista de Medicina

**Keywords:** dysphagia, dysphonia, aging, laryngeal nerves

## Abstract

**Introduction and aim:**

To carry out a morphometric analysis of myelinic fibers in laryngeal nerves aiming to identify quantitative changes as a result of aging. Study design: Clinical and experimental.

**Material and Method:**

A 1cm fragment was collected from the superior laryngeal nerves and recurrent laryngeal nerves taken from twelve male cadavers. The sample was divided into two groups: those aged below 60 years (Adult) and those aged 60 years or more (Elderly). The material was evaluated under light microscopy coupled with an image analysis system.

**Results:**

The total number of myelinic fibers from the superior laryngeal nerve was similar in both age groups; there was, however, a trend for a higher number of 1μm fibers in the adult group (p=0.0744). The adult group had a higher total number of myelinic fibers in the recurrent laryngeal nerve (p=0.0006), and this difference was seen in fibers with diameters betwee 1–3μm (p<0.007). The adult group had a higher total number of myelinic fibers in the laryngeal nerves (sum of superior laryngeal nerves and recurrent laryngeal nerves fibers) compared to the elderly group (p<0.0091).

**Conclusion:**

The total number of myelinic fibers in laryngeal nerves is higher for the group aged below 60 years.

## INTRODUCTION

The aging process alters the body, and much research has been done to define these changes in the upper aerodigestive tract; the larynx itself has been widely investigated. Most of the papers on the larynx have studied the mucosal layer that lines the vocal folds; various modifications have been described, such as: the reduced number of elastic fibers in the middle layer of the lamina propria;^1^ the reduced density of epithelial cells and the thickness of the lamina propria;^2^^,^^3^ the increased quantity of collagen in the lamina propria;^4^ morphological changes in elastic fibers located in the superficial layer of the lamina propria;^5^ and collagen fiber architectural derangement in the lamina propria.^6^

Studies on the laryngeal muscle system have revealed results that suggest the following: a reduced number of muscle fibers in the thyroarytenoid muscle, particularly slow contraction muscle fibers,^7^ and a reduced amount of those proteins that are responsible for muscle contraction.^8^ Based on electromyography studies, other authors have presented results that suggest denervation or axonal injury involving laryngeal motor control in the elderly, leading to altered contraction of laryngeal muscles.^9^

These vocal fold lining and laryngeal muscle changes are responsible for the quality of voice in elderly people, such as soprosity, hoarseness, loss of sonority and an altered fundamental frequency, which is increased in men and decreased in women.^10^^,^^11^

The aging process, besides the abovementioned voice changes, is associated with decreased pharyngeal and supraglottic sensitivity;^12^ this finding is considered one of the causes of dysphagia, aspiration, and repeat pneumonia in elderly patients due to diminished lower airway protection reflexes. Other changes in the elderly include delayed opening of the upper esophageal sphincter^13^^,^^14^ and reduced cricopharyngeus muscle tonus.^14^

The main function of the larynx is to protect lower airways during swallowing and to reduce glottic resistance during inspiration; as such, control of these functions depends on close integration between the sensory and motor systems. These include laryngeal receptors, general visceral afferent pathways, nervous system brainstem nuclei, special visceral efferent pathways and intrinsic laryngeal muscles.^15^

There are few studies on the effect of aging on laryngeal nerves;^16^, ^17^, ^18^, ^19^, ^20^ most of the papers on laryngeal innervation restrict themselves to purely anatomical issues.^21^, ^22^, ^23^, ^24^ Generally speaking, these papers state that the larynx is innervated by the vagus nerve through the following branches: the superior laryngeal nerve (SLN) and the recurrent laryngeal nerve (RLN).

The SLN is predominantly composed of smaller diameter afferent myelin fibers that course in the inner branch and are responsible for supraglottic and hypopharyngeal sensitivity,^17^^,^^18^^,^^20^^,^^25^^,^^26^ and by medium diameter efferent myelin fibers that course in the outer branch and that innervate the cricothyroideus muscle and part of the thyroarytenoid muscle.^23^^,^^25^^,^^26^ The RLN is composed by afferent and efferent myelin fibers and is responsible for subglottic sensitivity and for the innervation of the intrinsic laryngeal muscles,^25^^,^^27^^,^^28^ the superior region of the esophagus and part of the cricopharyngeus muscle.^29^^,^^30^

We undertook this study after having not found in literature any paper assessing quantitatively the myelin fibers of the superior and recurrent laryngeal nerves, and correlating these findings with aging.

The aim of this paper was to conduct a morphometric analysis of laryngeal nerve myelin fibers to verify quantitative changes ensuing from the aging process.

## METHOD

The Research Ethics Committee of our institution approved this research project, under number 0409/03. One-centimeter fragments were collected from the right and left SLNs and RLNs of 12 cadavers that had undergone autopsy between June 2003 and November 2004.

Male cadavers with no history of diseases such as diabetes, alcoholism, malignancies or sudden weight loss were assessed.^31^, ^32^, ^33^ The sample was divided into two groups, as follows: a group aged below 60 years (adult) composed by six cadavers, and a group aged 60 years or above (elderly) composed by six cadavers.

The SLN fragment was harvested 3cm from the thyrohyoid membrane, before the nerve division into an external and internal branch.^18^^,^^34^ The RLN fragment was harvested 4cm from the lower border of the cricoid cartilage; this site was chosen due to the increased possibility of harvesting all of the branches that innervate the larynx.^22^^,^^35^ The harvesting procedure included sectioning the nerve fragments cross-sectionally (perpendicular to the length axis of the nerve), making it possible to quantify the following morphometric measures: intraperineural cross-sectional area (representative of the number of myelin fibers) and the number and diameter of myelin fibers. Fragments were fixed in a glutaraldehyde solution at 2.5% plus paraformaldehyde at 2% in a sodium cacodylate 0.1 M buffer solution at pH 7.4 (modified by Karnovsky, 1965),36 post-fixed in osmium tetroxide at 2% in a sodium cacodylate 0,1 M buffer solution at pH 7.4, dehydrated in increasing concentrations of ethanol, and included in an Araldite 502□≪-type resin.

The material was sectioned by an ultramicrotome with glass knife to obtain ultrathin sections 0.3μm in width and stained toluidine blue at 1%. Sections were assessed in a light microscope coupled to an image analyzer‥

Morphometric evaluation was done in two steps:

A. Quantification of the intraperineural area: nerve images were digitized from the 5x lens to obtain a final magnification of 120x on the computer monitor. The intraperineural area is used to calculate the total number of nerve fibers based on a representative sample.

B. Quantification of the number and external diameter of myelin fibers: nerve images were digitized from the 40x lens to obtain a final magnification of 1,920x on the computer monitor. Four random fields per slide were assessed^37^ to count the number of fibers, to measure their diameters and to measure the representative field area; the perineural area was excluded. The area was assessed in each slide, varying from 6.1% to 25.9%. The total number of myelin fibers was estimated based on the total intraperineural area (obtained in the first step) and the number of fibers and the field area (obtained in the second step).

Myelin fibers projected over the lower and left lines that defined the field were excluded to avoid sampling errors.^38^ The smallest fiber diameter (the greatest distance perpendicular to the long axis of the myelin fiber) was chosen for measurements of myelin fibers that had elliptical or irregular perimeters.^39^

Analysis of variance (ANOVA) was used for the comparison of means (intraperineural area, density of myelin fibers/mm2 and the number of myelin fibers) between groups. The α significance level was 0.05. A value of p< 0.05 was considered significant.

## RESULTS

**Figure 1:**
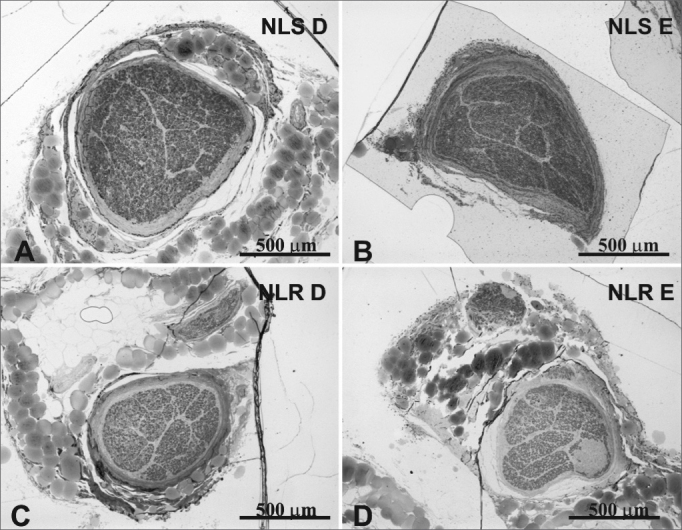
Typical photomicrograph of a cross-section of the right superior laryngeal nerve (A), the left superior laryngeal nerve (B), the right recurrent laryngeal nerve (C) and the left recurrent laryngeal nerve (D) in a 45-year-old subject. SLN D = right superior laryngeal nerve, SLN E = left superior laryngeal nerve, RLN D = right recurrent laryngeal nerve, RLN E = left recurrent laryngeal nerve. Staining was with toluidine blue.

**Figure 2:**
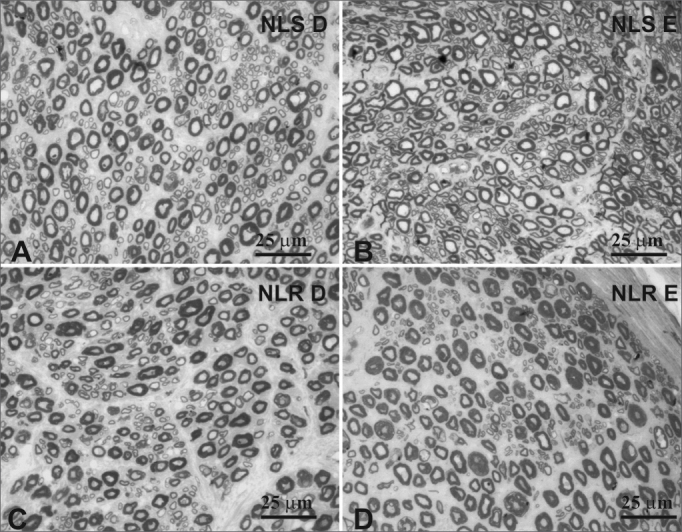
Typical photomicrograph of a cross-section of the right superior laryngeal nerve (A), the left superior laryngeal nerve (B), the right recurrent laryngeal nerve (C) and the left recurrent laryngeal nerve (D) in a 77-year-old subject. SLN D = right superior laryngeal nerve, SLN E = left superior laryngeal nerve, RLN D = right recurrent laryngeal nerve, RLN E = left recurrent laryngeal nerve. Staining was with toluidine blue.

**Figure 3:**
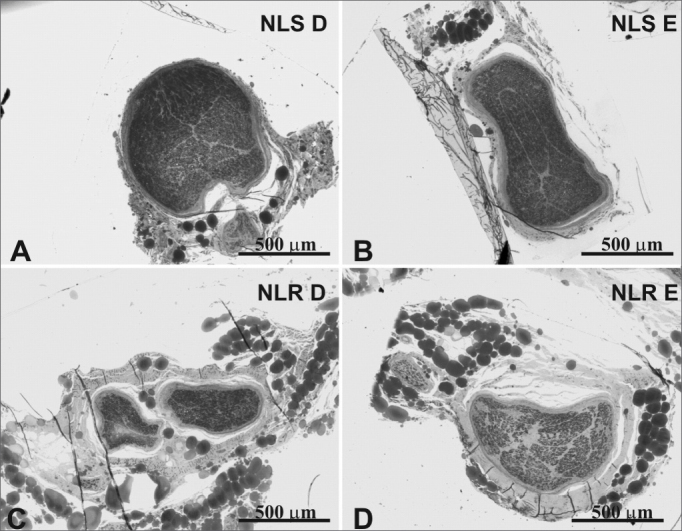
Typical photomicrograph of a field: right superior laryngeal nerve (A), the left superior laryngeal nerve (B), the right recurrent laryngeal nerve (C) and the left recurrent laryngeal nerve (D) in a 45-year-old subject. SLN D = right superior laryngeal nerve, SLN E = left superior laryngeal nerve, RLN D = right recurrent laryngeal nerve, RLN E = left recurrent laryngeal nerve. Staining was with toluidine blue.

**Figure 4:**
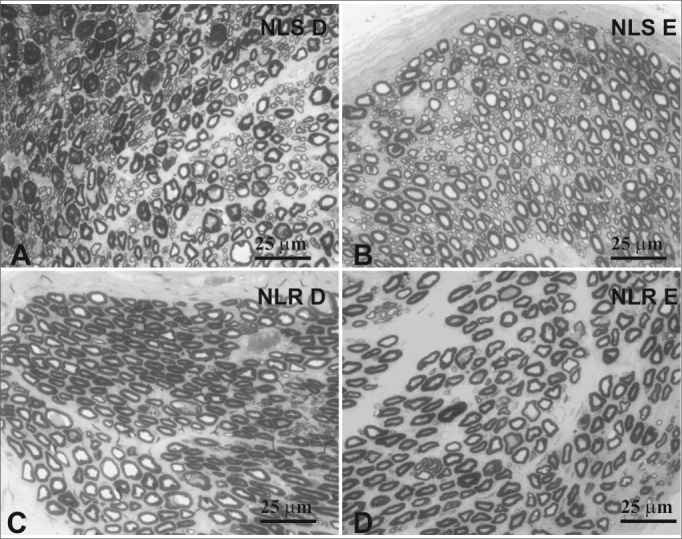
Typical photomicrograph of a field: right superior laryngeal nerve (A), the left superior laryngeal nerve (B), the right recurrent laryngeal nerve (C) and the left recurrent laryngeal nerve (D) in a 77-year-old subject. SLN D = right superior laryngeal nerve, SLN E = left superior laryngeal nerve, RLN D = right recurrent laryngeal nerve, RLN E = left recurrent laryngeal nerve. Staining was with toluidine blue.

**Figure 5:**
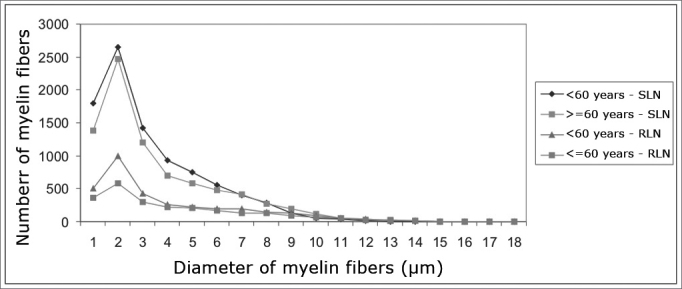
Adjusted mean profiles of the number of laryngeal nerve myelin fibers (superior laryngeal nerve - SLN, and the recurrent laryngeal nerve - RLN) in groups aged <60 years and ≥60 years according to fiber diameter.

The mean age of the adult group was 46.3 years; the mean age of the elderly group was 78.2 years. Descriptive data (mean and standard deviation) in each age group for the intraperineural area and the myelin fiber density/mm^2^ are presented on Table 1, Table 2. Table 3 presents descriptive data for SLN myelin fibers and Table 4 presents descriptive data for RLN myelin fibers; right and left sides have been groups together.Figure 1, Figure 2, Figure 3, Figure 4, Figure 5 provide easier visualization of the laryngeal nerve morphometric analysis. Figure 1, Figure 2, Figure 3, Figure 4 present typical laryngeal nerve photomicrographs of the cross-sectional area (lower magnification) and of a field (higher magnification) for each age group.

**Table 1:** Descriptive data on the laryngeal nerve intraperineural area (μm2) according to age groups.

Group		Area (μm2)			
		Right SLN	Left SLN	Right RLN	Left RLN
<60 years	Mean	450503	428805	232117	255281
	SD	88764	210044	43130	81333
	n	6	6	6	6
≥60 years	Média	434537	453259	195831	204816
	SD	156010	69006	38885	79591
	n	6	6	6	6

**Key:** SLN Dir = Right Superior Laryngeal Nerve; SLN Esq = Left Su perior Laryngeal Nerve; RLN Dir = Right Recurrent Laryngeal Nerve RLN Esq = Left Recurrent Laryngeal Nerve; SD = Standard deviation n = number of nerves

**Table 2:** Descriptive data on the density of laryngeal nerve myelin fibers/mm2 according to age groups.

Group		Density of myelin fibers/mm^2^			
		Right SLN	Left SLN	Right RLN	Left RLN
<60 years	Mean	21156	22335	14336	14129
	SD	4559	5698	3247	4780
	n	6	6	6	6
≥60 years	Mean	18628	19763	12692	11997
	SD	6104	4461	2772	3463
	n	6	6	6	6

**Key:** SLN Dir = Right Superior Laryngeal Nerve; SLN Esq = Left Superior Laryngeal Nerve; RLN Dir = Right Recurrent Laryngeal Nerve; RLN Esq = Left Recurrent Laryngeal Nerve; SD = Standard deviation; n = number of nerves

**Table 3:** Descriptive data on the number of right and left superior laryngeal nerve (SLN) myelin fibers according to the fiber diameter and the age group.

SLN		Diameter of myelin fibers (μm)															
		1	2	3	4	5	6	7	8	9	10	11	12	13	14	15	Total
<60 years	Mean	1795	2652	1428	928	750	554	395	287	130	54	36	9	0	1	0	9017
	SD	948	765	599	427	216	207	180	201	116	64	43	15	0	5	0	1692
	n	12	12	12	12	12	12	12	12	12	12	12	12	12	12	12	12
≥60 years	Mean	1387	2467	1205	701	580	485	417	278	191	110	50	29	9	7	2	7918
	SD	728	840	263	217	214	208	149	160	126	87	53	34	13	14	6	1624
	n	12	12	12	12	12	12	12	12	12	12	12	12	12	12	12	12

**Key:** SD = Standard deviation; n = number of nerves

**Table 4:** Descriptive data on the number of right and left recurrent laryngeal nerve (RLN) myelin fibers according to the fiber diameter and the age group.

RLN Group		Diameter of myelin fibers (μm)																		
		1	2	3	4	5	6	7	8	9	10	11	12	13	14	15	16	17	18	Total
<60 years	Mean	504	992	426	256	214	193	196	145	124	87	56	44	17	9	5	4	2	0	3276
	SD	208	248	127	99	93	104	102	81	86	82	52	42	21	15	10	9	4	0	383
	n	12	12	12	12	12	12	12	12	12	12	12	12	12	12	12	12	12	12	12
≥60 years	Mean	361	587	301	214	201	173	135	126	92	68	52	31	24	10	3	1	1	1	2381
	SD	207	365	154	142	152	95	76	76	58	58	46	41	37	19	7	3	3	2	669
	n	12	12	12	12	12	12	12	12	12	12	12	12	12	12	12	12	12	12	12

**Key:** SD = Standard deviation; n = number of nerves

There was no right to left difference in the SLN intraperineural area (p=0.9782) (Table 1, Figure 1, Figure 2); there was also no difference between age groups (p=0.9474). There was no right to left difference in the RLN intraperineural area (p=0.5322); there was also no difference between age groups (p=0.1426). The SLN, however, had a larger intraperineural area than the RLN in both sides, which was statistically significant (p<0.0001).

There was no right to left difference in the density of myelin fibers per mm2 in the SLN (p=0.5935) (Table 2, Figure 3, Figure 4); there was also no difference between age groups (p=0.2714). There was no right to left difference in the density of myelin fibers per mm^2^ in the RLN (p=0.7312); there was also no difference between age groups (p=0.2852). The SLN, however, had a higher myelin fiber density per mm2 than the RLN in both sides, which was statistically significant (p<0.0001).

There was no right to left difference in the number of myelin fibers in the SLN (p=0.8710). Right and left sides were grouped together for increased precision and a second analysis of variance was made to compare age groups and fiber diameters (Table 3, Figure 5). In this analysis no difference was found in the total number of myelin fiber between age groups (p=0.1188); in the nerve diameter analysis, however, there was a trend towards a higher number of 1μm fibers in the group aged below 60 years (p=0,0744).

There was no right to left difference in the number of myelin fibers in the RLN (p=0.9180). Right and left sides were grouped together for increased precision and a second analysis of variance was made to compare age groups and fiber diameters (Table 4, Figure 5). This analysis revealed that the total number of fibers in the group aged below 60 years was higher compared to the elderly group (p=0.0006); multiple comparisons of 1–3μm diameter myelin fibers showed a significant difference between age groups (p<0.007). There was no significant difference in the number and distribution of myelin fibers with diameters over 4μm.

The SLNs had more myelin fibers than the RLNs (p<0.0001) (Figure 5). Multiple comparisons revealed a significant difference in the diameter of 1–8μm myelin fibers, where SLNs had higher values. There was no statistical significance in the distribution of myelin fibers with diameters over 9μm. The adult group had a statistically significant higher total number of myelin fibers (sum of SLN and RLN fibers) compared to the elderly group (p<0.0091).

## DISCUSSION

The idea for this paper arose when we observed during medical work that many elderly patients presented voice disorders and swallowing difficulties (dysphonia and dysphagia). As there are few objective methods to assess these symptoms, we decided to approach the issue experimentally. Our aim was to evaluate the peripheral nervous system to detect morphometric alterations that would justify those complaints.

Few papers so far have analyzed age-related changes in laryngeal nerves. We found five papers dealing with this theme in the literature; two of these papers used animal models^16^^,^^17^ and three were studies on human beings.^2^, ^3^, ^4^, ^5^, ^6^, ^7^, ^8^, ^9^, ^10^, ^11^, ^12^, ^13^, ^14^, ^15^, ^16^, ^17^, ^18^, ^19^, ^20^ The SLN was studied in three papers^17^^,^^18^^,^^20^ and the RLN was investigated in two.^16^^,^^19^ We found no published paper that evaluated both laryngeal nerves (SLN and RLN) in humans and correlated findings with the aging process.

Using animal models to study age-related changes does not always bring results that can be applied to human beings, given that these animals are short-lived. This may explain why minimal alterations have been seen in studies on mice.^16^^,^^17^

Many published papers have described the laryngeal nerve morphometry; there were differences in methods, however, which have constrained the comparison of results. In our method we used previously reported parameters, such as the level at which SLNs were seccioned,^18^^,^^34^ the preparation of material and the morphometric analysis. The choice of site for harvesting the RLN was based on anatomical studies^22^^,^^35^ and on proof of altered morphometric features, depending on the section level.^40^, ^41^, ^42^

Our sample was composed of 12 male individuals with no history of diabetes, alcoholism or malignancies; these conditions would have altered the results, given the risk of peripheral neuropathy.^31^, ^32^, ^33^ Most of the papers on laryngeal nerve morphometry in human beings have used laryngeal nerve fragments obtained during laryngectomy in subjects diagnosed with laryngeal squamous cell carcinoma as their sample or control groups.^40^^,^^42^, ^43^, ^44^, ^45^, ^46^, ^47^, ^48^

We divided our sample into two groups; one was aged below 60 years and the other was aged 60 years or above. The mean age in the adult group was 46.3 years and the mean age in the elderly group 78.2 years; the mean age interval between groups was 31.9 years. One of the papers that correlated aging with RLN morphometry assessed a group of subjects aged over 60 years and a mean age of 75.1 years. Although this group was not compared with a younger group, the authors found an age-related decrease in the axonal area and perimeter.^19^

We found that the intraperineural area of the SLN was statistically significantly larger than that of the RLN. There was, however, no difference between the right and left area of the SLN or of the RLN in both age groups (Table 1, Figure 1, Figure 2). The mean area of the SLN in both sides was 0.44 mm^2^, which is similar to published mean values.^18^^,^^34^ The mean area of the RLN was 0.21mm^2^ on the right and 0.23mm^2^ on the left, which is slightly higher than the results published by Germain et al.^34^, probably due the fact that we sectioned the nerve 1cm more proximal to the vagus nerve. Repice^47^ reported that the cross-sectional are of the RLN was larger than that of the SLN, which runs contrary to our results and to other published data.^34^ As the RLN divides frequently close to the larynx, Repice^47^ may have included epineural areas, which would have led to higher than expected results.

The SLN had a higher density of myelin fibers per mm^2^ compared to the RLN, which was statistically significant. There was, however, no difference between right and left SLN and RLN myelin fiber density in both age groups; the younger group did show a trend towards higher values (Table 2, Figure 3, Figure 4). This finding was an indirect assessment method for laryngeal nerve morphometry. In other words, the SLN, which is composed predominantly by smaller diameter myelin fibers (sensorial fibers), had a statistically superior fiber density per mm^2^ compared to the RLN, which is composed by sensorial fibers (smaller diameter) and motor fibers (mid-sized diameter). Although not statistically significant, the adult group had a higher fiber density per mm^2^ than the elderly group, suggesting a predominance of smaller diameter fibers in the younger group. Other authors have compared the myelin fiber density per mm^2^ in right and left SLNs and RLNs and have reached similar results.^34^ Ravits et al.^45^ described a control group (mean age 60.7 years) that had a mean density of 9,420 fiber/mm^2^ in the RLN, which is lower than our values, but which may be related to a decrease in smaller diameter myelin fibers as a result of predisposing factors associated with malignancies, such as alcoholism or the paraneoplastic syndrome. Other authors have studied SLN fiber density per mm^2^ in normal subjects,^18^ having reached similar results to those in the current study, that is, a mean density of about 20,000 myelin fibers per mm^2^.

The myelin fiber distribution according to the diameter of the SLNs had a unimodal curve; 2μm predominated in both age groups. A higher frequency of smaller diameter fibers in the SLN had been demonstrated by many authors^17^^,^^18^^,^^20^^,^^25^^,^^26^^,^^43^^,^^47^^,^^49^ and is related to the primordially sensory function of this nerve. There was no right to left difference in the distribution of fibers according to their diameter (1μm to 15μm) in both age groups; there was also no difference between the age groups (Table 3, Figure 5). There was a trend towards a higher number of 1μm myelin fibers (p=0,074) in the younger group. Mortelliti et al.^18^ noted statistically significant differences in the total number of fibers and in 1μm and 2μm myelin fibers between two groups of different mean ages (mean age of the younger group was 23.8 years and the mean age of the elderly group was 76.2 years). Tiago et al.^20^ also found statistically significant differences not in the total number of fibers, but in 1μm to 2μm fibers; in this paper the mean age in the younger group was 39.6 years and the mean age of the elderly group was 71.2 years. In our study the mean age of the younger group was 46.3 years, which was higher than the other abovementioned studies; this did not facilitate comparisons, although it may suggest that the number of SLN myelin fibers starts to decrease after the fourth decade of life.

The distribution of myelin fibers according to the RLN diameter had a unimodal curve where 2μm fibers predominated (Table 4, Figure 5). Murtagh and Campbell^40^ published similar results to these and to the total number of fibers, which was similar to our findings for the elderly group. The distribution of myelin fibers in the RLN depends on the level at which the nerves were sectioned; as we move closer to the larynx, mid-sized diameter fibers become more frequent, particularly when assessing the anterior or muscle branch of the RLN.^25^^,^^44^^,^^46^^,^^48^^,^^49^ According to certain authors, smaller diameter fibers are located in the higher portion of the esophagus and the trachea, the subglottis^25^ and the cricopharyngeus muscle.^30^ In our sample there was no right to left difference in the distribution of fibers according to the diameter (1μm to 18μm) in both age groups. In the comparison of the distribution myelin fibers according to their diameter, the adult group showed a statistically significant higher total number of fibers than the elderly group in 1 μm to 3 μm diameter fibers. We found no published papers that described this finding.

A comparison between the number of SLN and RLN myelin fibers showed that the SLNs had over double the number of myelin fibers, which was statistically significant. This difference was seen in 1μm to 8μm diameter fibers (Figure 5). Other authors have published similar results,^34^ but with a lower total number of fibers; theses authors also did not describe at which fiber diameter these changes were seen. Furthermore, these authors used light microscopy and 5μm-thickness sections, which results in image overlapping and makes it impossible to count 1μm myelin fibers.

A decrease in the total number of laryngeal nerve myelin fibers in the elderly, particularly in smaller diameter (afferent) fibers, may be related to decreased laryngeal protection reflexes, making elderly patients more susceptible to aspiration and repeat pneumonia. Other changes may include altered regulating system of the intrinsic laryngeal muscle reflex tonus, which leads to laryngeal muscle flaccidness and voice changes that characterize presbyphonia.

## CONCLUSION

According to the morphometric analysis of laryngeal nerve myelin fibers in two separate age groups, it may be concluded that the group aged below 60 years presented a higher number of laryngeal nerve myelin fibers compared to the elderly group. This difference is evident in recurrent laryngeal nerves.
